# Prevalence and Classification of Scoliosis Among Female University Students in Saudi Arabia

**DOI:** 10.3390/healthcare13222894

**Published:** 2025-11-13

**Authors:** Marwan M. A. Aljohani, Yasir S. Alshehri, Reda S. Eweda

**Affiliations:** 1 Department of Physical Therapy, College of Medical Rehabilitation Sciences, Taibah University, Madinah 42353, Saudi Arabia; yshehri@taibahu.edu.sa (Y.S.A.); rawedah@taibahu.edu.sa (R.S.E.); 2 Orthopedic Physical Therapy Department, Faculty of Physical Therapy, Cairo University, Giza 12613, Egypt

**Keywords:** epidemiology, idiopathic scoliosis, functional scoliosis, spinal deformity, screening

## Abstract

**Highlights:**

**What are the main findings?**

- Scoliosis was present in 35.7% of female university students: 26.2% had functional scoliosis and 9.5% had structural scoliosis.
- Functional scoliosis was associated with habitual sitting posture, leg-length discrepancy, and age, whereas structural scoliosis was associated with habitual sitting posture, family history, and back pain.

**What is the implication of the main finding?**

- Screening should differentiate functional from structural scoliosis and include routine assessment of posture and leg-length discrepancy.
- Targeted ergonomic education and early physiotherapy may help correct reversible postural curves in functional scoliosis and support symptom management in individuals with structural scoliosis.

**Abstract:**

**Background/Objectives:** Although screening for scoliosis is common among adolescents, little is known about its subtypes and their correlations in young adults. This study aimed to investigate the prevalence and classification of scoliosis (functional vs. structural) among female university students in Saudi Arabia and to examine associated factors. **Methods:** A cross-sectional study was conducted with 263 female students at Taibah University. Screening was performed using Adam’s forward bending test and a scoliometer. Data on age, body mass index, academic year, hand dominance, habitual sitting posture, backpack carriage method, leg-length discrepancy, painful conditions, and family history of scoliosis were collected. The association between scoliosis subtypes and contributing factors was analyzed using descriptive statistics, chi-square test, and Spearman’s correlation. **Results:** Scoliosis was identified in 94 students (prevalence, 35.7%). Of these, 26.2% had functional scoliosis, whereas 9.5% had structural scoliosis. Functional scoliosis was significantly associated with sitting posture, leg-length discrepancy, and age (*p* < 0.05), whereas structural scoliosis was associated with family history, habitual sitting posture, and painful conditions (*p* < 0.05). **Conclusions:** The high prevalence and differing profiles of scoliosis subtypes underscore the need for routine postural screening in universities. Early identification and ergonomic education may help in selecting appropriate targeted interventions for individuals with functional or structural scoliosis.

## 1. Introduction

Scoliosis is a three-dimensional spinal deformity characterized by lateral curvature, vertebral rotation, and abnormalities in the sagittal plane [1]. It primarily affects adolescents, with adolescent idiopathic scoliosis (AIS) being the most common [2]. Although the exact cause is unclear, genetic predisposition, hormonal factors, and biomechanical influences have been suggested as potential contributing factors [3]. Globally, a large U.S. study of college-age women (mean, ~19.7 years) found clinical scoliosis in ~12% of young adults. Which is a substantially higher prevalence than that reported in younger adolescents [4]. Approximately 0.5–4% of adolescents are estimated to have scoliosis, with the pooled prevalence in the 1–3% range [5]. A recent meta-analysis of school-age children reported idiopathic scoliosis in approximately 1.7% of cases, with a pooled prevalence of approximately 1.12% in boys and 4.51% in girls [6]. Approximately 80% of scoliosis cases are structural, with congenital and neuromuscular causes accounting for the majority of the remaining cases [7]. Various factors, including age, sex, genetic predisposition, and occupational posture, are associated with scoliosis progression [8]. Notably, scoliosis is more frequent in females, with reported female-to-male ratios of approximately 1.5:1 to 3:1 in adolescence: this disparity widens with more severe curves [9]. This female predominance and the potential for curvature progression make scoliosis a significant clinical concern in young people.

Scoliosis is broadly classified into two main categories: structural and functional. Structural scoliosis is defined as a fixed spinal deformity characterized by a three-dimensional curvature of the spine. It includes lateral deviation and axial rotation, that does not resolve with changes in posture or positioning [10]. In contrast, functional scoliosis refers to reversible spinal curvature caused by external or postural factors, such as muscle spasms, limb-length discrepancies, or habitual asymmetric postures, and is not associated with vertebral rotation [11]. The hallmark of functional scoliosis is correction upon elimination of the underlying cause or through changes in body position. Differentiating these types is clinically important, particularly for early detection and management. Prevalence studies that distinguish structural and functional scoliosis may aid in early identification and intervention, potentially preventing the progression of spinal deformities and their associated complications.

Studies in Saudi Arabia have reported varying scoliosis prevalence but rarely distinguished curve types [12,13,14,15]. For instance, an earlier Riyadh study involving participants aged 11–13 found AIS in 2.5% of participants, with significant curves (≥5°) present in 0.8% [13]. A recent Riyadh school survey found that approximately 29.4% of 12–18-year-olds screened positive for suspected AIS [15], and a similar study in Abha reported a 19.1% prevalence among male adolescents [12]. However, none of these studies explicitly differentiated between the structural and functional cases. Instead, they reported only the overall scoliosis or the prevalence of AIS. Consequently, the relative burden of functional curves in the Saudi population remains unknown.

Scoliosis screening typically involves Adam’s forward bending test and a scoliometer, a non-invasive tool known for its simplicity and clinical utility [16,17]. The Adam’s forward bending test visually detects spinal rotation and rib humps [18], whereas a scoliometer quantifies trunk rotation [19]. An angle of trunk rotation (ATR) ≥ 5° warrants follow-up, and ≥ 7° suggests the need for radiographs as it correlates with a Cobb angle ≥ 10°, the diagnostic threshold for scoliosis [20,21]. Combining these tools enhances detection while minimizing radiation exposure [8].

Structural AIS is predominantly driven by intrinsic factors [2]. It typically emerges around the pubertal growth spurt and shows strong female predominance, with female-to-male ratios as high as 10:1 in severe cases. Genetic factors are central and family clustering is common, with up to 50% of idiopathic cases reporting a positive family history [22]. Twin studies have suggested heritability as high as 38% [23]. Anthropometric factors have also been implicated; adolescents with AIS tend to be taller and have a lower body mass index (BMI) than their unaffected peers [24,25]. For instance, Zhou et al., 2022, reported that underweight status (BMI ≤ 20) significantly increased the odds of scoliosis in Chinese adolescents [24]. Clinically, untreated structural scoliosis can lead to chronic back pain and postural dysfunction [25]. In contrast, functional scoliosis is associated with extrinsic biomechanical factors [5,26,27,28]. One well-documented cause is leg-length discrepancy (LLD), which induces a compensatory lumbar curve [27]. Raczkowski et al. [27] demonstrated that applying a shoe lift resolved 83.7% of such cases within two weeks. Functional scoliosis is distinguished by its reversibility, which typically resolves upon lying down or correcting the underlying biomechanical trigger and lacks vertebral rotation [17].

Despite these internationally established associations, no prior studies in Saudi Arabia have systematically differentiated structural from functional scoliosis. In addition, no study analyzed their respective correlates in young adult Saudi populations. Existing Saudi research often reports the overall scoliosis prevalence or suspected AIS but does not account for postural factors such as LLD and habitual sitting posture. This gap limits the ability to develop targeted screening or intervention strategies based on the scoliosis type. The current study aimed to address this gap by estimating the prevalence of structural and functional scoliosis among female Saudi university students and identifying the factors associated with each scoliosis type. Therefore, this study aimed to (1) determine the prevalence of scoliosis and classify it into structural and functional types among female Saudi university students, and (2) examine the demographic, hereditary, and biomechanical factors associated with each scoliosis subtype. We hypothesized that structural scoliosis would be associated with intrinsic and nonmodifiable factors such as family history and painful conditions, while functional scoliosis would be linked to modifiable biomechanical factors such as habitual sitting posture and LLD.

## 2. Materials and Methods

### 2.1. Subjects

This cross-sectional study included 263 female students at Taibah University. The sample size was determined a priori using an estimated scoliosis prevalence of 19%, a 95% confidence level and 5% margin of error [14]. Using the standard methods for prevalence studies, the minimum required sample size was determined to be 236. To ensure sufficient statistical power and account for potential data loss or exclusion (approximately 11%), the sample size was increased to 263 participants. Data were collected between March and June 2024. The researchers advertised the study to all female students through campus posts and social media, inviting interested participants. Those who agreed to participate received written and verbal explanations about the study’s purpose, procedures, and requirements, as well as signed informed consent forms before undergoing assessments. As for the inclusion criteria, female students from Taibah University participated in this study. The exclusion criterion was the inability to follow simple instructions, such as standing still for longer than 10 min or performing forward trunk bending. Demographic data on age, college years, year of study, weight, height, hand dominance, backpack carriage method, painful conditions, and family history were collected. This study was approved by the Ethics Committee at Taibah University (approval no. CMR-PT-2024-033).

### 2.2. Procedures of Assessment

#### 2.2.1. Inspection

The first step in the assessment of scoliosis is postural inspection. This includes inspection of a standing student from behind and visual evaluation of asymmetries in the shoulders, scapulae, waistline, distance of the arms from the trunk, and balance of the head [19]. The participants were then asked to stand still for 10 min, and body alignment was screened in the posterior, anterior, and lateral views [29]. Additionally, LLD was assessed by measuring the distance from the anterior superior iliac spine to the medial malleolus in supine position.

#### 2.2.2. Adam’s Forward Bending Test

Adam’s forward bending test is the principal screening method used to detect trunk asymmetry (e.g., rib prominence) [29]. This test examines the back and identifies the rotational component of scoliosis that causes rib prominence, as the rib cage rotates with the spine. Adam’s forward bending test is a sensitive clinical examination compared to the Cobb angle, but its accuracy varies based on the examiner’s skill, curve location, and curve magnitude [29]. For thoracic curves ≥ 10°, sensitivity is 74–84% and specificity is 78–93%, while for thoracic curves ≥ 20°, sensitivity is 92–100% and specificity is 60–91%. For lumbar curves ≥ 20°, sensitivity is 73% and specificity is 68%. For curves ≥ 40°, sensitivity is 83% and specificity is 99%. Overall, Adam’s forward bending test is an effective screening tool, with intra- and interobserver reliability ranging from very good to excellent in several studies [29].

In this study, the test was performed in a standing position [18]. Female students were instructed to stand upright, tie their hair up, and wear customized backless t-shirts to provide a full view of their backs. They were then asked to bend forward at the waist while the examiner assessed the symmetry of the back from the posterior and lateral views. The spinal curvature and rotation were subsequently quantified using a scoliometer.

Adam’s forward-bending test was used to classify participants as having structural or functional scoliosis. Structural scoliosis was defined as a posterior rib/trunk prominence that persists during forward flexion, whereas functional scoliosis was defined as an apparent standing asymmetry that resolves with forward flexion [30].

#### 2.2.3. Measurement of Inclination Angle

A manual scoliometer (Baseline Metal Scoliosis Meter, Fabrication Enterprises, White Plains, NY, USA) was used in this study to assess spinal curvature. It is a handheld device commonly used when scoliosis is suspected and is placed over the spinous processes at the level of the maximum paraspinal prominence [31]. The scoliometer measures the angle of trunk rotation, which can be used to estimate the Cobb angle more accurately using radiographic imaging [31]. Multiple studies have demonstrated that scoliometers have very good to excellent intra- and inter-observer reliability [29]. The validity of the scoliometer when compared with the gold standard Cobb angle on radiographs has been found to be fair to very good [32]. In this study, we used a cut-off angle of 5 degrees or more on the scoliometer to define scoliosis. This threshold was selected based on prior validation studies demonstrating that it provides high sensitivity and specificity for detecting Cobb angles of 10° and 20°, making it appropriate for screening purposes [32].

The students were asked to reveal the upper part of the back, bend forward slowly, and stop when the shoulders were levelled with the hips. We also viewed the students from both front and back before measuring with a scoliometer. We adjusted the height of the student’s bending position to the level at which the deformity of the spine was most pronounced, gently rested the scoliometer on the skin, and noted its direction and degree. The measurements in this study were taken by an experienced physical therapist with more than 15 years of clinical practice in musculoskeletal and spine disorders.

### 2.3. Statistical Analysis

Descriptive statistics, including means and standard deviations, were calculated for continuous variables such as age, weight, height, and BMI. Frequencies and percentages were computed for categorical and continuous variables, including the presence of scoliosis, type of scoliosis, habitual position, LLD, backpack carriage preference, family history of scoliosis, hand dominance, academic year, college, and presence of painful conditions. Spearman’s correlation was used to investigate the association between the scoliosis type (i.e., functional and structural) and continuous data. The chi-square test of independence was used to examine the association between the scoliosis type and categorical variables. Cramér’s V test was used to assess the strength of the relationship. Statistical significance was set at *p* < 0.05. All statistical analyses were performed using SPSS software version 25 (IBM Corp., Armonk, NY, USA).

## 3. Results

### 3.1. Participants

This study analyzed the prevalence and characteristics of scoliosis in a sample of 263 female university students. The participants’ characteristics are summarized in Table 1.

### 3.2. Prevalence of Scoliosis

Scoliosis was identified in 35.7% of the sample (Table 2). The prevalence of scoliosis was highest among fourth-year students (37.2%), followed by second-year (30.9%), third-year (19.1%), and first-year (12.8%) students. Among those with scoliosis, 94.7% were right-handed, and 14.9% reported a positive family history. The age distribution of scoliosis cases showed that most affected students were between 19 and 22 years old, accounting for 98.8% of all cases (19 years = 22.3%, 20 years = 25.5%, 21 years = 25.5%, 22 years = 25.5%). Smaller proportions were observed among students aged 23 years (6.4%) and 25 years (1.1%). Painful conditions were identified in 16.7% of scoliosis cases. Most curves were left-sided (17.4%) or right-sided (13.6%), with few cases exhibiting a combined or atypical pattern. The thoracic region was the most common site of involvement (78.7%), followed by the thoracolumbar (17%) and lumbar (4.3%) regions. With respect to habitual posture, 56.7% of students with scoliosis maintained a neutral sitting position, 30% inclined to the right, and 13.3% inclined to the left. Backpack carriage was primarily on the right side (63.5%), with fewer students carrying their backpacks on the left side (25.5%) or using a neutral approach (11%).

### 3.3. Functional and Structural Scoliosis Subgroups

Among students diagnosed with scoliosis, 69 had functional scoliosis (26.2%), whereas 25 had structural scoliosis (9.5%) (Table 2). Pain was reported by 40% of students with functional scoliosis and 64% of students with structural scoliosis (Table 3). A neutral habitual sitting posture was reported by 49.3% in the functional scoliosis group and 32% in the structural group, whereas a right-inclined posture was noted in 32% and 40% of students, respectively. Backpack carriage on the right side was reported by 58.0% of students with functional scoliosis and 68% of those with structural scoliosis. A family history of scoliosis was reported by 11.6% of students with functional scoliosis and 24% of those with structural scoliosis. Curve location differed between the groups; functional scoliosis was primarily thoracic (97.1%), whereas structural scoliosis was distributed across the thoracic (20%), thoracolumbar (64%), and lumbar (16%) regions. The age distribution of students with functional scoliosis showed that most cases occurred between 19 and 22 years of age, representing 94.8% of the functional subgroup (19 years = 21.7%, 20 years = 23.2%, 21 years = 17.4%, 22 years = 30.4%). In contrast, structural scoliosis was also concentrated in this same age range, accounting for 92% of cases (19 years = 24.0%, 20 years = 32.0%, 21 years = 24.0%, 22 years = 12.0%), with fewer cases at age 23 (8.0%).

Separate chi-square analyses were performed within the functional and structural scoliosis subgroups to explore the associations with categorical variables. In the structural scoliosis group, significant associations with a family history of scoliosis (χ^2^(1) = 6.5, *p* = 0.02, Cramér’s V = 0.184), the presence of painful conditions (χ^2^(1) = 6.4, *p* = 0.016, Cramér’s V = 0.182), and habitual sitting position (χ^2^(2) = 11.7, *p* = 0.004, Cramér’s V = 0.246). These results suggest a potential link between structural scoliosis and hereditary, symptomatic factors, and postural factors. No statistically significant associations were observed between year of study, hand dominance, or backpack carriage method (all *p* > 0.05). In the functional scoliosis group, only habitual sitting position was significantly associated (χ^2^(2) = 6, *p* = 0.04, Cramér’s V = 0.159), indicating that postural behavior may play a role in functional scoliosis. No significant associations were found between family history, painful conditions, and academic year (all *p* > 0.05).

In the functional scoliosis group, scoliosis type showed a moderate positive correlation with LLD (r = 0.49, *p* < 0.001, 95% CI [0.387, 0.581]). A weak but statistically significant correlation was also found with age (r = 0.133, *p* = 0.04, 95% CI [0.006, 0.256]). No significant correlations were found between the scoliosis type and weight, height, or BMI (*p* > 0.05). In the structural scoliosis group, no statistically significant correlations were observed between the scoliosis type and any of the continuous variables (*p* > 0.05).

## 4. Discussion

Nearly one-third (35.7%) of the 263 female university students screened exhibited scoliosis. Specifically, 26.2% were functional and 9.5% were structural. Functional scoliosis was significantly associated with a positive habitual sitting position, age, and LLD. On the other hand, structural scoliosis was significantly associated with a positive family history, the presence of back or neck pain, and habitual sitting position. The findings of this study are important, as they show the distinct factors associated with functional and structural scoliosis, facilitating an understanding of different factors that affect each type. Therefore, this study can guide targeted screening, prevention, and management strategies tailored to each subtype.

### 4.1. Prevalence of Scoliosis

Our overall structural scoliosis prevalence of 9.5% is substantially higher than the 2–4% AIS prevalence typically reported in adolescents [2,9]. This is plausibly reflecting our older, all-female university sample and clinical screening. Consistent with greater asymmetry on visual screening in late adolescence/early adulthood, the Utah college women study reported 12% with visible lateral deviation [4], and a Riyadh school survey found 29.4% suspected cases [15]. Recent systematic review and meta-analysis estimated pooled AIS prevalence globally (e.g., 1.7–3.1% in children/adolescents [6]), but heterogeneity is substantial [6,33]. National estimates span 0.05–16.67%, highest in Romania, and city-level school screenings in China report 11.1%, 13.4%, and 14.0% [33]. This suggests that mild or progressive curvatures may continue to develop during late adolescence and early adulthood. This high prevalence of structural scoliosis in our study might be explained by the older age of participants. Moreover, as university students, participants spend long hours sitting and studying, which may increase cumulative postural asymmetry. Combined with the high rate of positive family history (>30%), these factors may have contributed to the elevated risk of curve progression [33]. In our sample, more than 70% of scoliosis cases were functional. In contrast, radiographic screening studies confirm that only structural scoliosis (Cobb angle ≥ 10° with rotation) has a prevalence of approximately 2–4% [2,9]. One possible explanation for this discrepancy is that functional scoliosis is posture dependent and reversible; hence, it is rarely observed in radiography-based epidemiology. This suggests that many students in our study likely exhibited mild functional scoliosis. In other words, university students may frequently present with postural spinal asymmetries that, while not fulfilling the diagnostic criteria for idiopathic scoliosis, constitute notable deviations from normal spinal alignment and may warrant clinical attention.

Our data revealed that nearly all the functional curves were localized in the thoracic region (97.1%), likely reflecting the effects of habitual thoracic slouching or lateral Inclination. On the other hand, the structural curves were most frequent across thoracolumbar (66.7%), followed by the thoracic (22.2%), and lumbar (11.1%) regions. This distribution aligns with the established patterns of idiopathic scoliosis, in which structural curves frequently involve the thoracolumbar region [2]. The localization of functional curves in the thoracic region underscores the impact of prolonged asymmetrical sitting, which can induce reversible spinal deviations. Furthermore, recent evidence suggests that insufficient physical activity and prolonged sedentary behavior may contribute to postural imbalance and spinal asymmetry [33]. Thus, our findings support the notion that lifestyle-related factors are key contributors to functional scoliosis and highlight the importance of early ergonomic and behavioral interventions.

### 4.2. Factors Associated with Scoliosis

Analysis of factors associated with scoliosis supports the distinction between functional and structural scoliosis. In structural scoliosis, significant associations have been observed between family history and back/neck pain, consistent with prior evidence linking AIS to a hereditary predisposition and symptomatic burden [1,3,33]. This result agrees with a recent meta-analysis in that identified family history and poor sitting posture as risk factors for scoliosis across 51 studies involving over 1.6 million children and adolescent [33]. Another study suggested that abnormal posture is strongly associated with suspected scoliosis [5]. A possible explanation is that postural asymmetry with the predisposing genetic factors may affect paraspinal muscle activation pattern and vertebral loading, leading to structural adaptation over time which may contribute to scoliosis development [34,35]. On the other hand, a five-year retrospective study of Brazilian (mean age, 14 years) found no significant association between postural habits and the presence of scoliosis [36]. This discrepancy may be attributed to age differences across studies. The younger participants in that cohort likely had less cumulative exposure to asymmetric postural loading, whereas our university-aged sample (mean age, 20 years) may have experienced longer durations of poor sitting posture, sufficient to induce adaptive spinal changes. Therefore, our findings emphasize the importance of sustained postural asymmetry over time as a potential factor influencing the transition from functional to structural scoliosis. Significant associations were observed between back pain and structural scoliosis, consistent with prior evidence linking scoliosis and back pain [15,37,38,39]. The mechanism may include asymmetric paraspinal muscle activation, vertebral rotation altering facet joint mechanics, and compensatory postural changes leading to discomfort [34,35,39]. In contrast, previous research suggested that the relationship between pain and scoliosis is multifactorial [38,39,40]. These findings suggest that both biomechanical and psychosocial factors may play a role in back pain in individuals with structural scoliosis. Therefore, comprehensive approach to managing back pain in individuals with structural scoliosis is warranted. On the other hand, no significant associations were found between behavioral or anthropometric variables, including academic year, hand dominance, backpack side, LLD, age, weight, height, or BMI, and structural scoliosis. This finding supports the understanding that structural scoliosis is predominantly driven by intrinsic genetic and multifactorial factors [22]. However, the interpretation of findings related to structural scoliosis should be made cautiously, as the small sample size may have limited the statistical power to detect true associations.

In contrast, functional scoliosis was significantly associated with modifiable factors, particularly habitual sitting posture and LLD, emphasizing its biomechanical origin and potential reversibility [27,41,42,43]. Structural scoliosis, by comparison, was significantly linked to habitual sitting posture, but not to LLD, highlighting a shared but divergent biomechanical pathway. These contrasting patterns underscore the need for subtype-specific assessment and intervention strategies. The observed association between functional scoliosis and LLD suggests that pelvic asymmetry and prolonged asymmetric loading may contribute to compensatory spinal curvature in functional cases. Prior studies confirm that even small LLD discrepancies can alter load distribution and trigger such adaptations [27,41,42,43]. However, evidence directly examining posture-related and functional scoliosis remains limited. Therefore, our finding extends current knowledge by showing that these biomechanical influences persist into young adulthood and may contribute to early structural spinal changes. This supports the model proposed by Hawes and O’Brien [44], which suggests that chronic asymmetrical loading and postural imbalance may, over time, lead to fixed vertebral and discal changes characteristic of structural scoliosis. Prospective longitudinal studies are warranted to confirm this progression and to evaluate whether early correction of postural asymmetry can prevent structural transformation.

In our study, age was significantly associated with functional scoliosis but not with structural scoliosis. This finding suggests that the prevalence of functional scoliosis increases with age, possibly due to the cumulative effects of poor posture or prolonged asymmetrical loading during university years. To date, no previous research has specifically explored this association, highlighting a gap in the literature. Future research is needed to confirm our findings and further investigate the underlying mechanisms.

### 4.3. Clinical Implications

Scoliosis detected in our student study may have clinically relevant consequences. There is growing evidence that spinal curvature can contribute to musculoskeletal pain and affect quality of life [39,45,46,47]. Previous studies have shown that spinal curvature is frequently accompanied by pain and functional limitations in young adults [25]. In the present study, many students with scoliosis reported experiencing back and neck pain. This is consistent with a recent large-scale Saudi study in adolescents; nearly 45% of those screened had back pain and those with suspected scoliosis scored significantly worse on the self-image and mental health subscales of the SRS-22 quality of life questionnaire [15]. Uncorrected functional scoliosis can lead to muscle fatigue, asymmetric strain, and pain [27]. Additionally, the psychological impact should not be overlooked; young women may experience diminished body image or confidence knowing that they have an uneven spine [15,39,45,46,47]. Moreover, aesthetic concerns and body image distress are well documented among young women with trunk asymmetry [48]. These findings collectively highlight the importance of early identification and management of mild scoliosis. From a clinical standpoint, interventions such as physiotherapy, targeted exercises (e.g., scoliosis-specific core stabilization), and patient education on posture can alleviate pain symptoms and possibly improve cosmetic alignment [49,50]. Furthermore, early recognition of scoliosis in young adults can guide targeted management strategies for posture-related back pain, potentially reducing the reliance on generalized or symptomatic treatment.

In our study, scoliosis subtypes were classified based on the persistence of rib prominence during the Adam’s forward bend test, designating structural scoliosis when asymmetry remained fixed and functional scoliosis when it resolved. Although this clinical methodology is effective for practical application, it may not comprehensively encapsulate the underlying etiological complexities. For instance, in the early or mild stages of idiopathic scoliosis, truncal rotation and the resultant rib hump may predominantly originate from asymmetrical rib growth rather than solely from vertebral rotation [51]. This perspective suggests that rib cage asymmetries may assume a primary role in the initiation of the deformity, potentially accounting for suboptimal postoperative corrections in structural cases and emphasizing the utility of rib-specific metrics, such as the rib index, in prospective screening protocols [51]. In the current study, where functional scoliosis was often thoracic and posture-related, integrating such assessments could refine differentiation and guide more targeted interventions, though the Adam’s forward bend test effectively identified reversible versus fixed deformities for practical management.

### 4.4. Limitations and Future Directions

This study has several limitations. It focused solely on female students from one university, limiting generalizability. Future studies should include males, multiple institutions, and diverse age groups. Although scoliosis screening was based on clinical examination, radiographic confirmation was not obtained. This was partly due to the fact that functional scoliosis, which is posture-related, may not be detectable on radiographs. However, the lack of radiographs may have inflated the apparent prevalence of scoliosis. The cross-sectional design of this study limits the ability to establish cause-and-effect relationships between scoliosis and its associated factors. Therefore, it cannot be determined whether variables such as posture, LLD, or physical activity contribute to scoliosis development or result from existing spinal curvature. The limited sample size of the structural scoliosis subgroup (*n* = 25) substantially reduces the statistical power to identify subgroup-specific risk factors and renders multivariate regression analyses unstable and inappropriate. Longitudinal studies are needed to assess whether mild curvatures improve or worsen over time and to examine college-related risk factors such as sitting duration, physical activity, backpack load, and device use. Although the sample included students from diverse academic disciplines, we acknowledge that those enrolled in the physiotherapy program may have greater awareness of musculoskeletal conditions and family history which potentially influence their responses.

## 5. Conclusions

This study identified a high prevalence of scoliosis among female Saudi university students, predominantly functional in type. Both functional and structural scoliosis were significantly associated with habitual sitting posture. However, LLD and age were specific to functional scoliosis, while family history and back pain were specific to structural scoliosis. These findings emphasize the need to distinguish scoliosis subtypes during screening and support incorporating postural assessments and ergonomic education into university health programs to prevent long-term complications. While these associations offer important insights, findings related to structural scoliosis should be interpreted with caution. Future research should include longitudinal and multi-center studies involving both male and female participants to confirm the observed prevalence, explore potential curve progression beyond adolescence, and enhance the generalizability of these findings.

## Figures and Tables

**Table 1:** Demographic data (mean ± SD).

Age (years)	20 ± 1.38
Weight (kg)	55.39 ± 12.03
Height (m)	1.56 ± 0.067
BMI (kg/m^2^)	22.72 ± 4.75

**Table 2:** Sample characteristics (*n* = 263).

Variable	*N*	%	Scoliosis Cases (*n*)	Total Scoliosis Cases (%)
***Scoliosis***				
Present	94	35.7	-	-
Not present	169	64.3	-	-
***Year***				
First	45	17.1	12	12.8
Second	88	33.5	29	30.9
Third	56	21.3	18	19.1
Fourth	74	28.1	35	37.2
***Dominant hand***				
Right	249	94.7	88	94.7
Left	14	5.3	5	5.3
***Type of scoliosis***				
Functional	69	26.2	-	-
Structural	25	9.5	-	-
***Painful conditions with scoliosis***				
Yes	44	16.7	**-**	**-**
No	49	18.6	**-**	**-**
***Directions of scoliosis***				
LT	43	17.4	**-**	**-**
LT thoracic RT lumbar	7	1.5	**-**	**-**
RT	35	13.6	**-**	**-**
RT thoracic LT lumbar	9	2.6	**-**	**-**
***Locations of scoliosis***				
Lumbar	4	4.3	**-**	**-**
Thoracic	74	78.7	**-**	**-**
Thoracolumbar	16	17	**-**	**-**
***Family history***				
Yes	27	10.3	14	14.9
No	236	89.7	80	85.1
***Habitual position***				
Inclined to LT	35	13.3	20	21.3
Inclined to RT	79	30	32	34
Neutral	149	56.7	42	44.7
***Backpack carriage***				
Right	167	63.5	57	60.6
Left	67	25.5	24	25.5
Neutral	29	11	13	13.8

**Table 3:** Frequency by type of scoliosis.

Variable	Category	Functional (*n*)	Structural (*n*)
Family history	No	61	19
	Yes	8	6
Site of scoliosis	Lumbar	0	4
	Thoracic	67	5
	Thoracolumbar	2	16
Backpack carriage	Left	18	6
	Neutral	11	2
	Right	40	17
Habitual position	Inclined to the left	13	7
	Inclined to the right	22	10
	Neutral	34	8
Painful conditions	No	40	9
	Yes	29	16
Dominant hand	Left	4	1
	Right	65	24
Year	1st	7	5
	2nd	21	8
	3rd	14	4
	4th	27	8

## Data Availability

The data presented in this study are available on request from the corresponding author. The data are not publicly available due to respondents’ privacy.
